# The Microbial Nitrogen Cycling, Bacterial Community Composition, and Functional Potential in a Natural Grassland Are Stable from Breaking Dormancy to Being Dormant Again

**DOI:** 10.3390/microorganisms10050923

**Published:** 2022-04-28

**Authors:** Bikram K. Das, Satoshi Ishii, Linto Antony, Alexander J. Smart, Joy Scaria, Volker S. Brözel

**Affiliations:** 1 Department of Biology and Microbiology, South Dakota State University, Brookings, SD 57006, USA; bikram.das@sdstate.edu; 2 Water and Climate Institute, University of Minnesota, St. Paul, MN 55108, USA; ishi0040@umn.edu; 3 Biotechnology Institute, University of Minnesota, St. Paul, MN 55108, USA; 4 Veterinary and Biomedical Sciences Department, South Dakota State University, Brookings, SD 57006, USA; linto.antony@jacks.sdstate.edu (L.A.); joy.scaria@sdstate.edu (J.S.); 5 Department of Natural Resource Management, South Dakota State University, Brookings, SD 57006, USA; alexander.smart@sdstate.edu; 6 Department of Biochemistry, Genetics and Microbiology, University of Pretoria, Pretoria 0004, South Africa

**Keywords:** prairie, grassland, nitrogen cycle, soil bacterial community

## Abstract

The quantity of grass-root exudates varies by season, suggesting temporal shifts in soil microbial community composition and activity across a growing season. We hypothesized that bacterial community and nitrogen cycle-associated prokaryotic gene expressions shift across three phases of the growing season. To test this hypothesis, we quantified gene and transcript copy number of nitrogen fixation (*nifH*), ammonia oxidation (*amoA*, *hao*, *nxrB*), denitrification (*narG*, *napA*, *nirK*, *nirS*, *norB*, *nosZ*), dissimilatory nitrate reduction to ammonia (*nrfA*), and anaerobic ammonium oxidation (*hzs*, *hdh*) using the pre-optimized Nitrogen Cycle Evaluation (NiCE) chip. Bacterial community composition was characterized using V_3_-V_4_ of the 16S rRNA gene, and PICRUSt2 was used to draw out functional inferences. Surprisingly, the nitrogen cycle genes and transcript quantities were largely stable and unresponsive to seasonal changes. We found that genes and transcripts related to ammonia oxidation and denitrification were different for only one or two time points across the seasons (*p* < 0.05). However, overall, the nitrogen cycling genes did not show drastic variations. Similarly, the bacterial community also did not vary across the seasons. In contrast, the predicted functional potential was slightly low for May and remained constant for other months. Moreover, soil chemical properties showed a seasonal pattern only for nitrate and ammonium concentrations, while ammonia oxidation and denitrification transcripts were strongly correlated with each other. Hence, the results refuted our assumptions, showing stability in N cycling and bacterial community across growing seasons in a natural grassland.

## 1. Introduction

The microbial role in Nitrogen (N) cycling within naturally sustaining grasslands or prairies is complex [1] and subject to fluctuations in environmental conditions [2]. In North America, grasslands once formed the largest vegetative province, covering about 162 × 10^6^ Ha [3]. Today, over 99% are cropland, with some areas converted back to livestock pasture over time [4]. The productivity of such soil systems rely on the diversity of soil microbes [5]. However, studies show that intensive agricultural practices cause a decrease in microbial diversity [6,7]. Therefore, to understand how microbial nitrogen conversions shape ecological productivity, there is a need to study the in situ dynamics of the resident microbial community. Specifically, during maximum biological activity, especially between the phases of breaking dormancy to the beginning of harsh winters.

Large proportions of soil bacteria are heterotrophs requiring a supply of organic carbon for growth and metabolic activity [8]. An important source of carbon for these microbes is the root exudates [9,10]. Although degraded root materials are an alternative carbon and nitrogen source, they are very recalcitrant and can be used only when broken down to simpler molecules [11]. However, root exudates are easily accessible to soil microbes [12]. At the beginning of the growing season, root exudates are high [13,14], followed by a decline towards the end of the season [15]. In addition, younger plants have been reported to release large amounts of root exudates compared to mature plants [16], evidentially seen to affect nitrification and nitrogen fixation [10]. Therefore, a seasonal trend is expected in soil microbial community composition [17] and functional potential due to shifts in soil chemical properties [18].

Microbially catalyzed N cycling is highly acknowledged and studied [19,20], especially after the recognition of the damaging environmental effects of N-based fertilizers [21]. N-fixing microbes are strongly associated with N-poor rhizospheres because they need labile carbon sources. Moreover, the expression of *nifH*, widely used as a marker for nitrogenase, differs seasonally [22,23] in response to plant activity and seasonal variations. Furthermore, a study by Langarica-Fuentes et al. showed that the addition of artificial root exudates increased nodulation, nitrogen fixation, and bacterial denitrification [24]. Similarly, all nitrogen cycle processes would concomitantly be affected due to fluxes in the bacterial activity.

An upper-mid-west natural prairie, Sioux Natural Prairie, is a self-sustaining natural ecosystem. After the end of long and harsh winters, the prairie revives, housing vibrant biological activities supported by diverse plant communities. The source of N for plants in the natural ecosystem is mainly due to microbial N fixation. Here, we sought to characterize the bacterial role in nitrogen interconversions over a growing season. We hypothesized that nitrogen cycle-associated prokaryotic gene expressions shift across three phases of the growing season, spring, summer, and early fall, which spanned from May to October. In addition, we predicted that there should be changes in the soil community and their functional potential, specifically for N-cycling, from the breaking of dormancy to the onset of the winter season. The majority of grasses in this study site are perennial. Perennial grasslands like the tallgrass prairie have about a 50% root turnover (meaning they die and eventually break down and get incorporated as soil organic matter) [25]. We excluded the winter season as the ground is frozen, and there is comparatively no plant activity. To test these hypotheses, we quantified the copies of the N cycle functional genes and their respective transcripts in prairie soil collected from a single transect 100 m long. A pre-optimized assay was used to assess the N-cycle genes using a Nitrogen Cycle Evaluation (NiCE) chip [26], an integrated fluidic chip for carrying out high throughput qPCR. The processes tracked included nitrogen fixation (*nifH*), ammonia oxidation (*amoA*, *hao*, *nxrB*), denitrification (*narG*, *napA*, *nirK*, *nirS*, *norB*, *nosZ*), dissimilatory nitrate reduction to ammonia (*nrfA*), and anaerobic ammonium oxidation (*hzs*, *hdh*). Furthermore, we compared the bacterial community using 16S rRNA gene sequencing, and functional predictions were made using PICRUSt2-v2.4.1 [27], an efficient method to generate microbial functional inferences from 16S rRNA sequence data.

## 2. Materials and Methods

### 2.1. Sample Sites

The Sioux Prairie Preserve near Colman, South Dakota, is preserved by The Nature Conservancy, dedicated to the preservation of natural areas for ecological studies. Samples were taken from six sites, which were 20 m apart from each other along a randomly obtained transect between 44°2′8.5″ N, 96°47′5.2″ W and 44°2′5.6″ N, 96°47′6.9″ W. The first site was selected randomly, and a transect was taken in a random direction. Hence, a non-probability sampling method was employed. Soil samples were first taken when the ground was thawed during May 2018 and then monthly till October (six time points). Three soil cores were taken to a depth of 5–7 cm using a soil corer of 2.5 cm diameter. Samples were placed in sterile plastic bags and kept in the dark and on ice till processing in the laboratory a few hours later. To determine soil composition, pH, nitrate, ammonia, total nitrogen (by Kjeldahl method [28]), potassium, and extractable phosphorous (by Olsen method [29]), sub-samples collected from the study site were analyzed by the South Dakota State University (SDSU) Soil Testing Laboratory using standard protocols. Plant diversity at each location was determined by foliar analysis. A 1 m square frame was placed around each site three times, and the foliar cover was estimated to the nearest 1% of each plant species.

### 2.2. Extraction of Nucleic Acids

Soil cores were ground and mixed to homogeneity, and 1 g of sample was supplemented with 2 mL of LifeGuard Soil Preservation Solution (Qiagen, Hilden, Germany) in sterile 15 mL conical tubes to protect mRNA from degradation. Samples were mixed by vigorous shaking, centrifuged at 7500× *g* for 10 min, and excess preservative was removed. RNA was extracted using Qiagen’s RNeasy PowerSoil Total RNA Kit according to the manufacturer’s protocol. RNA samples (20 µL) were incubated with 2 µL 10× DNA suspension buffer and 1 µL of TURBO DNase (Thermo Fisher, Waltham, MA, USA) for 20 min at 37 °C to remove any residual DNA. The reaction was stopped by adding 2 µL of DNase inactivation reagent. After a 5 min incubation at room temperature, tubes were centrifuged at 10,000× *g* for 15 min. The supernatant containing the purified RNA was transferred to new tubes. DNA was obtained using the RNeasy PowerSoil DNA Elution Kit to extract microbial DNA from the same spin column. DNA and RNA were quantified spectrophotometrically using a NanoDrop 2000 Spectrophotometer (Thermo Scientific), and fluorometrically using the Qubit™ dsDNA BR Assay Kit and Qubit™ RNA BR Assay Kit in a Qubit^®^ 3.0 Fluorometer (Thermo Fisher). The extracts were preserved at −80 °C until further processing. Absence of bacterial DNA in RNA extracts was verified by PCR using the universal primers 27F (AGA GTT TGA TCM TGG CTC AG) [30] and 518 R (GTA TTA CCG CGG CTG CTG G) [31] to amplify the V_1_ to V_3_ region of bacterial 16S rRNA genes. To determine whether DNA contained potential inhibitors of PCR, the same PCR was performed using undiluted and 10- and 100-fold diluted DNA extract as a template. PCR was carried out using 0.6 μL dNTPs (10 mM), 0.6 μL (10 μM) of each Primer, and 2.4 μL (25 mM) MgCl_2_, 0.2 μL (5 U/μL) Taq polymerase (NewEngland BioLabs, Ipswich, MA, USA), 3 μL (10×) PCR Buffer, and nanopore filtered water up to 30 μL reaction volume. The PCR reaction condition included initial denaturation at 95 °C for 4 min, 30 cycles at 95 °C for 30 s, 50 °C for 45 s, and 72 °C for 1 min, and a final elongation at 72 °C for 10 min.

### 2.3. cDNA Preparation

The first strand of complementary DNA (cDNA) was synthesized by using the PrimeScript^TM^ RT reagent kit (Takara Bio, Kusatsu, Shiga, Japan). The reaction mixture (10 µL) contained 2 µL of 5× PrimeScript Buffer, 0.5 µL of Enzyme mix, 0.5 µL of Random hexamers (100 µm), 5 µL of RNase-free water, and 2 µL of RNA template. The tubes were incubated at 37 °C for 15 min, followed by 80 °C for 5 s. The cDNA was stored at −80 °C.

### 2.4. Standards Preparation for the NiCE Chip

Nitrogen Cycle Evaluation (NiCE) chip uses a very small volume of samples to run quantitative polymerase chain reaction (qPCR) for numerous assays simultaneously. A 48 × 48 integrated fluidic circuits (IFC) chip can help carry 2304 reactions in a single run. This allows rapid quantification of targeted genes in total DNA extracts. In addition, the use of standards ensures the validity of the qPCR run [26]. Standards were prepared by mixing 2.5 µL each of 30 different gBlock or plasmid solutions (2 × 10^9^ copies/μL) (Table S1) and 25 µL of nuclease-free (Diethyl Pyrocarbonate (DEPC)—treated) water (final volume = 100 µL). The final concentrations of each gBlock were 5 × 10^7^ copies/µL. The gBlock mixture was serially diluted to make 5 × 10^6^, 5 × 10^5^, 5 × 10^4^, 5 × 10^3^, 5 × 10^2^, 5 × 10^1^, and 5 copies/µL in 8-well PCR tube strips. The last well was filled with 100 µL of nuclease-free water for a no-template control (NTC).

### 2.5. Specific Target Amplification for the NiCE Chip

Specific target amplification (STA) was conducted to increase the amount of templates for qPCR on the NiCE chip [32]. Both DNA/cDNA samples and the standards were subject to the STA reaction. The STA reaction is a multiplex PCR completed with all primers with a small number of PCR cycles [33]. Each STA reaction mixture included 4 µL of 2× TaqMan™ PreAmp Master Mix (Thermo Fisher Scientific, Waltham, MA, USA), 2 µL of STA primer pool (0.2 µM each primer), and 2 µL of the template. The STA reaction was completed using a Veriti Thermal Cycler (Applied Biosystems, Waltham, MA, USA) with the following condition: 95 °C for 10 min, followed by 14 cycles at 95 °C for 15 s and 60 °C for 4 min, and cooling to 4 °C. To remove unreacted primers, 1 µL of exonuclease I buffer and 0.5 µL Exo I (New England Biolabs, Ipswich, MA, USA) were added to each STA reaction product and incubated at 37 °C for 30 min. The reaction was terminated by incubating the plate at 85 °C for 20 min. Reaction mixtures were diluted to 40 µL by adding 30.5 µL of water and stored at −20 °C. For conventional qPCR, the products from STA reactions were further diluted 10 times.

### 2.6. NiCE Chip Amplicon Quantification

Two different 48 × 48 integrated fluidic circuits, IFCs (Fluidigm, South San Francisco, CA, USA), were used for the NiCE chip amplicon quantification, one for the DNA and another for RNA samples. Forty-one assay pre-mixes were prepared by mixing 2.5 µL of 2× Assay loading reagent (FLuidigim), 1 µL of 20 µM primer pair mix (Table S2), and 1.5 µL of DNA suspension buffer in wells of a 96-well plate. Sample pre-mix was also prepared in a 96-well plate. Each well contained 4 µL of 2× SsoFast EvaGreen Supermix with low ROX (BioRad, Hercules, CA, USA), 0.4 µL of 20× DNA Binding Dye (Fluidigm), and 3.6 µL of Exo 1-treated and diluted STA product.

Five microliters of each of the assay pre-mix and sample pre-mix were pipetted into respective inlets of the 48.48 IFC and mixed by using the IFC controller MX (Fluidigm) according to the manufacturer’s instructions. The qPCR was completed using the Biomark HD system (Fluidigm) with the following conditions: hot start at 98 °C for 120 s followed by 40 cycles of 98 °C for 5 s, 50 °C for 20 s, and 72 °C for 30 s. A melting curve cycle was added to the run, which had an initial temp of 60 °C raised to 98 °C at a rate of 1 °C/3 s. Furthermore, ROX was used as a passive dye. Negative control samples (extraction blanks and no template controls) were negative for all, and the GE Fast 48 × 48 PCR + Melt v2.pcl program was initiated.

The qPCR data from the NiCE chip runs were evaluated using Fluidigm Real-Time PCR Analysis software (Version 4.5.2). The thresholds of the amplification curves were adjusted to represent all the sample runs for each assay. Next, the R^2^ values of each calibration curve were generated, and any assay with an R^2^ value less than 0.95 was considered invalid and dismissed from further analysis. Furthermore, the melting curve and amplification plot for each run was evaluated to select valid results. As a result, 25 assays were selected for the downstream analyses (Supplementary Data S3).

### 2.7. 16S rRNA Gene Sequence-Based Community Profiling

Illumina sequencing of the 16S rRNA gene amplicon pools was performed using standard procedures. The V_3_-V_4_ regions of the bacterial 16S rRNA gene were PCR amplified using Illumina 16S Amplicon primers with overhang adaptors (forward: 5′ TCGTCGGCAGCGTCAGATGTGTATAAGAGACAGCCTACGGGNGGCWGCAG 3′ and reverse: 5′ GTCTCGTGGGCTCGGAGATGTGTATAAGAGACAGGACTACHVGGGTATCTAATCC 3′). Sequencing libraries were prepared using the Nextera XT library preparation kit and dual indexing method (Illumina, Inc., San Diego, CA, USA) as per the manufacturer’s protocol. After purification, quantification, and normalization, all 36 libraries were pooled and run on an Illumina Miseq platform using paired-end V2 chemistry. The raw data is available on NCBI’s Sequence Read Archive (SRA) database under SRA: SRP358531; BioProject: PRJNA803487 (https://www.ncbi.nlm.nih.gov/bioproject/PRJNA803487 Uploaded 6 February 2022). Microbiome taxonomic profiling was performed using QIIME 2 [34], and functional analysis was performed using PICRUSt2 [27]. The OTU clustering was performed on the paired-end sequence reads were denoised using the DADA2 pipeline [35]. The taxonomic analysis was performed by generating a feature classifier [36] extracted using forward and reverse primers from the Greengenes database version 13.5 [37]. A recent paper in the International Journal of Systematic and Evolutionary Microbiology (IJSEM) outlined name changes to all bacteria phyla [38]. As our data were processed using the Greengenes database, we chose to reflect the currently used phylum names. The sequencing data from site 2 for May was very low despite the high nucleic acid concentrations. Therefore, it was excluded from the downstream analysis of the bacterial community.

### 2.8. Data Analysis

RStudio [39] and R [40] were used along with multiple packages for statistical analysis and data visualization. The data was first sorted using the “*dyplr*” package [41] and then plotted using the package—“*ggplot2*” [42]. Statistical analyses were also performed using “*agricolae*” [43], “*DescTool*” [44], “*psych*” [45], “*ggpubr*” [46], “*vegan*” [47], and “*tidyverse*” [48] in R Studio. A regression analysis was performed using the “*car*” package [49]. A multidimensional scaling method was used to translate the pairwise distances of the copy numbers by months across the growing season and sites. Furthermore, dual-line graphs were generated to study the fluctuation of the copy numbers across the growing season.

The diversity of bacteria from the OTU table was further explored and evaluated using the Microbiomeanalyst webtool [50,51]. The pre-set basic filtering technique was used to include all OTUs for an overview of bacterial diversity across the growing season for each site. Then, Inkscape was used to edit the generated graph. The same webtool was used to generate Clusters of Orthologous Groups of Protein (COGs) from the metagenomic predictions or KO estimate from PICRUSt2.

## 3. Results

We found that the N-cycle activity and bacterial community composition of Sioux prairie soil were largely unchanged across a growing season, except for May. Gene and transcript quantities related to N cycling remained constant from May through October. Furthermore, the functional potentials of the bacteria derived through PICRUSt2 were estimated, revealing a uniform distribution of the functional capabilities across the seasons. The predicted prevalence of genes related to dissimilatory and assimilatory nitrate reduction was high across the seasons. In contrast, the functional genes for secondary metabolite production and motility were very low.

### 3.1. Heterogeneity of Prairie

The prairie landscape is undulating and heterogeneous. To determine soil composition and plant diversity, we sampled six equally spaced sites along a random 100 m transect of the Sioux Prairie monthly from May to October. Soil composition varied little across the transect. All sites had clay-loam soil, whereas site 2 had a silty-clay-loam texture (Figure S1). The concentrations of Nitrate-N and Ammonium varied across the sampling months (*p* < 0.01); however, Olsen-Phosphorus, Potassium, Total Kjeldahl Nitrogen (TKN), and pH did not vary significantly (Figure S2). Plant diversity determined by the Foliar cover method varied across the transect, indicating heterogeneity. Sites 1–4 were dominated by cool-season grasses and were devoid of legumes, whereas sites 5 and 6 contained a few legumes (Figures S3 and S4). Site 6 hosted more diverse flora, while the lowest-lying Site 3 was the least diverse and dominated by *Phalaris arundinacea* (reed canary grass), which thrives in water-logged conditions. In contrast, the other five sites were rich in *Bromus inermis* (smooth bromegrass), while *Solidago missouriensis* (Missouri goldenrod) predominated Site 5. Therefore, the prairie was inherently multifarious in soil composition and flora. Our experimental approach sought to capture microbial N cycle activity across this landscape. To determine the physiochemical properties of the soil, key chemicals in the soil were tested. As the growing season advanced, nitrate increased (R = 0.51, *p* < 0.05, Figure S2A) whereas ammonium decreased (R = −0.52, *p* < 0.05, Figure S2E). Furthermore, the pH did not change significantly across the seasons, but varied across the sampling sites (Figure S2D). Similarly, the changes in the amount of Olsen–Phosphorus, Potassium, and TKN did not vary significantly across the time points.

### 3.2. Prairie N Cycle Functional Genes and Transcripts across the Growing Season

**Figure 1:**
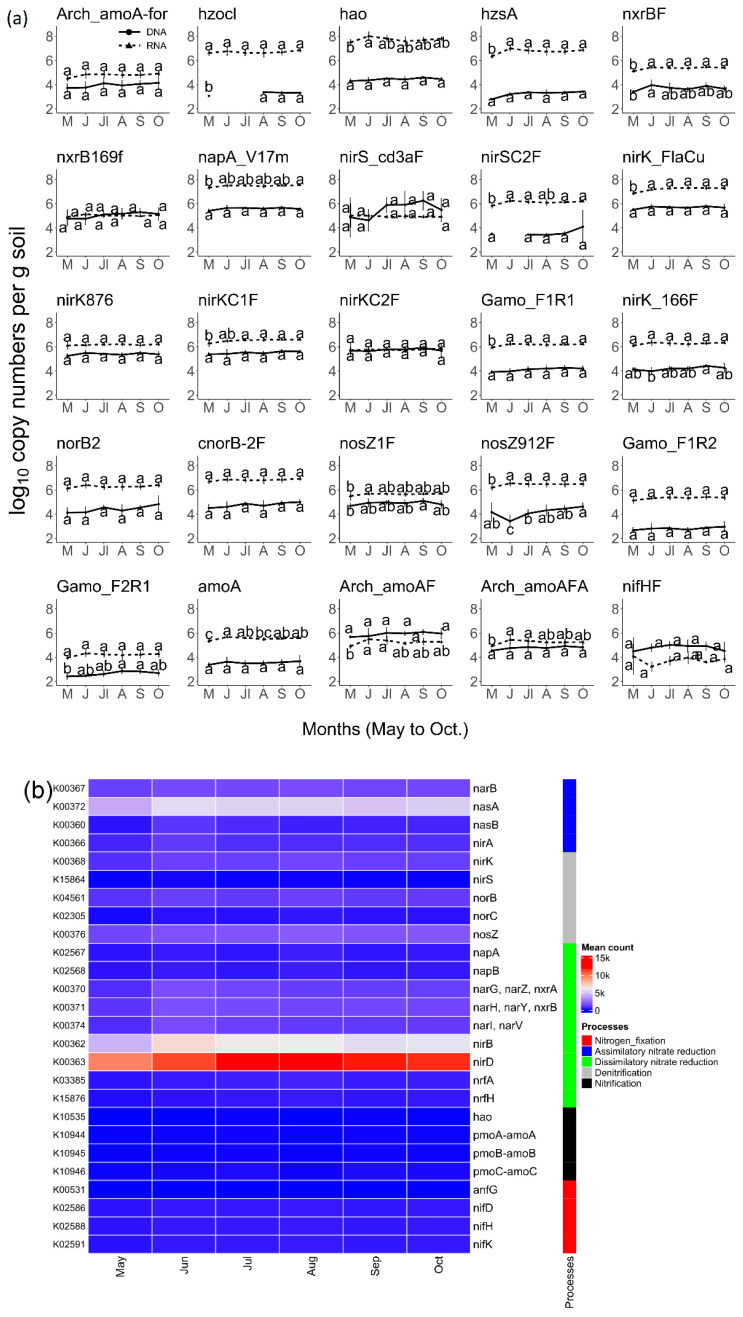
N cycle genes and transcript counts did not change across seasons. (**a**) Copy numbers of nitrogen cycle gene and gene expression levels across the growing season determined using the NiCE chip and expressed as log10. Letters on the line graph represent groups from Tukey HSD test [ANOVA, *p* < 0.05]; (**b**) heatmap of selected orthologs associated with nitrogen cycle, derived from PICRUSt2 analysis.

To quantify nitrogen cycle genes and their transcripts in a natural prairie system, we used 41 pre-optimized primer sets (Table S1) for the estimation of the gene copies and transcript quantities on different NiCE chips—one for the genomic DNA and another for the total RNA. The R^2^ values obtained for the amplification of G-Block solutions (Table S2) for each primer pair were used as a quality control measure. Twenty-seven of the forty-one assays yielded standard curves with R^2^ values > 0.95, and data with lower R^2^ values were not used for further analyses. Two of these assays were related to bacterial and archaeal 16S rRNA genes. The remaining N-cycle-related gene copies and their respective quantities obtained from the chip data followed a horizontal trend across the sampling time points (Figure 1a), with some differences in the *nosZ* gene. Comparatively, the transcript quantities for genes associated with nitrogen fixation (*nifH*), archaeal nitrification (*amoA* measured by the Arch_amoA assay), and bacterial denitrification (*nirS* measured by the cd3aF assay, *nosZ* clade I measured by the nosZ1F assay) were either lower than or the same as respective gene copies. In contrast, the RNA quantities were high compared to DNA or gene copies for other valid assays (Figure 1a). Based on the Kruskal–Wallis test, log_10_-transformed gene copy numbers for the genes *hao*, *nxrB*, *nirK*, *amoA* (γ-proteobacteria), *nirK*, and *nosZ*, measured by respective assays (Table S1), were different in at least one of the six months (*p* < 0.05). Furthermore, the transcript copies for the genes *hzs*, *nxrB*, *napA*, *nirS*, *nirK*, *amoA* (γ-proteobacteria), *nosZ*, and the ammonia oxidation genes were different for at least one of the six months (*p* < 0.05). Tukey HSD test results showed that the transcript quantities for May were significantly different from those for other months for several of the target N cycle genes (*hao*, *hzsA*, *nxrBF*, *napA*, *nirS*, *nirK*, *amoA*, and *nosZ*). The analysis of a subset of KEGG ortholog (KO) numbers associated with nitrogen metabolism elucidated that the predicted gene copies did not vary across seasons. The relative count obtained from PICRUSt2 prediction of the genes related to dissimilatory nitrate reduction (*nirB* and *nirD*) and assimilatory nitrate reduction (*nasA*) were high, but the predicted mean counts were similar across all months. The generally stable numbers of nitrogen cycle genes indicated the presence of a stable soil microbial community that is not very responsive to changes in seasons or other temporal factors such as nitrate (Figure S2A) or ammonia concentrations (Figure S2E). The transcript quantities were in accordance with functional predictions of nitrogen metabolism genes by PICRUSt2.

### 3.3. Bacterial Activity in the Nitrogen Cycle

**Figure 2:**
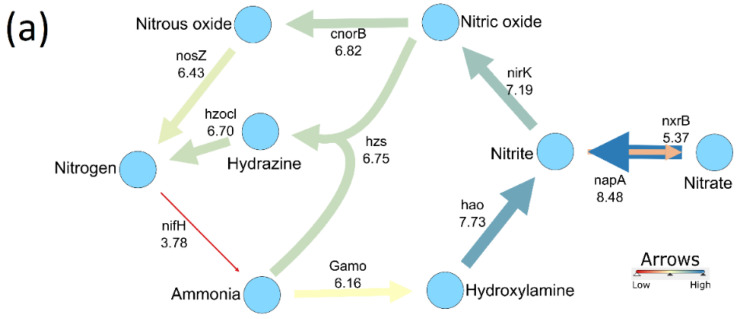

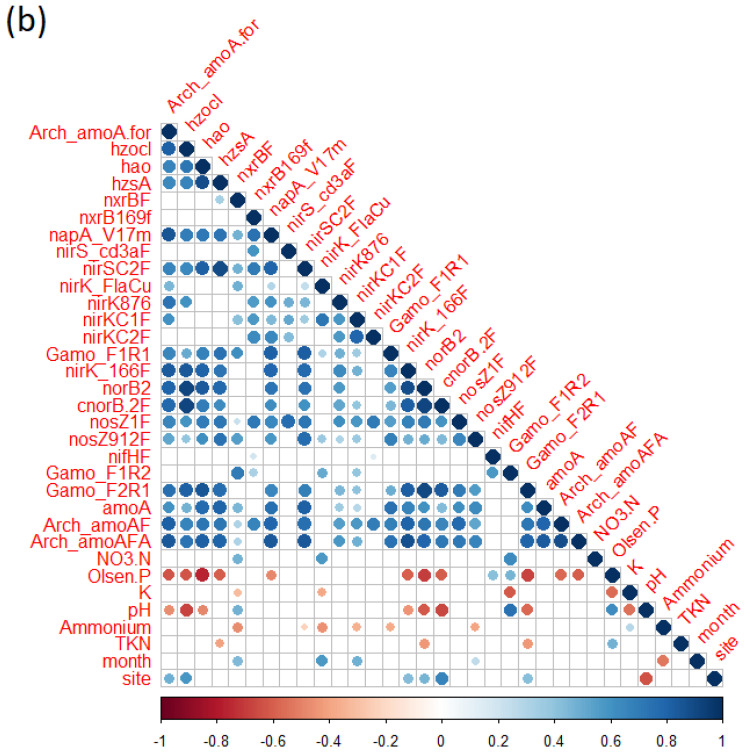
Interrelationships of genes of the nitrogen cycle. (**a**) Average transcript levels of the 25 genes are indicated by thickness of arrows connecting intermediates. The processes and corresponding genes were nitrate reduction [*napA*], denitrification [*nirK*, *cnorB*, and *nosZ*], nitrification [*Gamo*, *hao*, and *nxrB*], anammox [*hzsA* and *hzocl*], and nitrogen fixation [*nifH*]. No reactions were included for assimilatory nitrate reduction, and the result for DNRA [*nrfA*] for nitrite to ammonia conversion was invalid because R^2^ value was <0.95 for the standard curve. (**b**) Correlations between the 25 gene transcripts and chemical soil properties derived by Spearman correlation showing only significant correlations (*p* < 0.01).

A bacteria-driven N cycle with important biochemical transformations and their relationship with soil properties would aid in the understanding of the bacterial role in the prairie system. For that, we constructed a network graph (Figure 2a) where the edge strengths were determined by using overall averages of the transcript quantities. Furthermore, in cases where multiple gene products represented the catalysis of the same reaction, the highest value was used to represent the conversion. The nitrogen cycle (Figure 2a) obtained, and all key reactions, were facilitated through similar mRNA levels. Quantities of the bacterial nitrogen fixation gene, *nifH* (red arrow), were very low. In comparison, correlations between the transcript quantities and soil chemical properties were evaluated. Some of the soil chemical properties and gene transcript quantities showed a negative correlation, whereas several N cycle gene quantities were positively correlated (*p* < 0.01) (Figure 2b). The transcript quantities associated with archaeal ammonia oxidation (*amoA* measured by Arch_amoA assay), nitrate reduction (*napA* measured by napA_V17m assay), hydroxylamine dehydrogenase (*hao*), ammonia oxidation (γ-proteobacteria), nitric oxide reduction (*cnorB*), and nitrite reduction (*nirK*) are negatively correlated with phosphorus (Olsen P) content and pH of the soil. In contrast, transcript quantities for those processes were strongly correlated with each other. Overall, the bacteria sustained the flow of interdependent processes of the N cycle. However, *nifH* transcript quantities showed a weak positive correlation with phosphorus (Olsen P) content, ammonia oxidation (γ-proteobacteria), nitrite reduction (*nirK*), and nitrate reduction (*nxrB*). Therefore, there is evidence supporting a nitrogen cycle that was largely in synchrony (Figure 2b).

### 3.4. N-Cycle Gene Copy and Transcript Numbers Are Both Similar across Seasons

**Figure 3:**
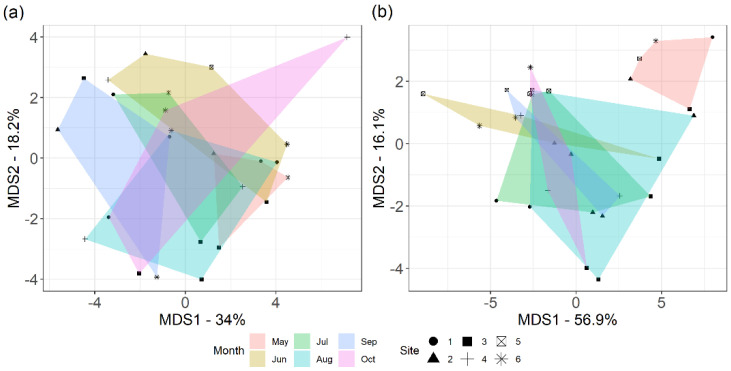
Multidimensional scaling (MDS) of N–cycle–related genes using Euclidean distance for 25 selected assays. (**a**) DNA copies; (**b**) transcript quantities.

To understand how gene copy and transcript quantities varied across timepoints, we performed multidimensional scaling (MDS) for both the gene copy and transcript copies of N-cycle genes (Figure 3). The Euclidean distance of the bacterial genes and their respective quantities resulted in overlapping clusters; that is, the gene presence was not distinct for any of the time points (Figure 3a). However, transcript quantities for May had well separated Euclidean distances from data points of other months (Figure 3b). The replicates for the seasons had very dissimilar gene copies and were far apart. However, the transcript quantities for the replicates from Sites 5 and 6 clustered together and away from other sites. The transcript quantities from Site 3 also differed.

### 3.5. N-Cycle Genes Are Strongly Transcribed except nifH

**Figure 4:**
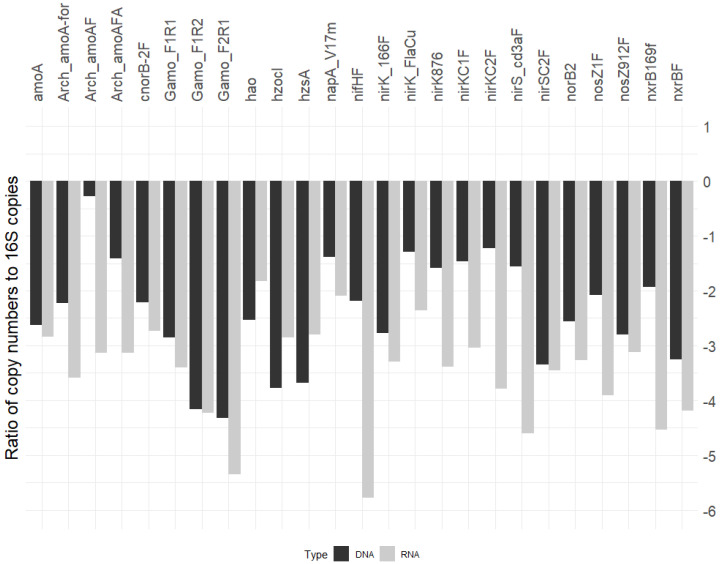
Proportion of N–cycle gene copy numbers and transcript quantities to total bacterial/archaeal biota as measured by bacterial and archaeal 16S rRNA gene qPCR, expressed as log_10_.

**Figure 5:**
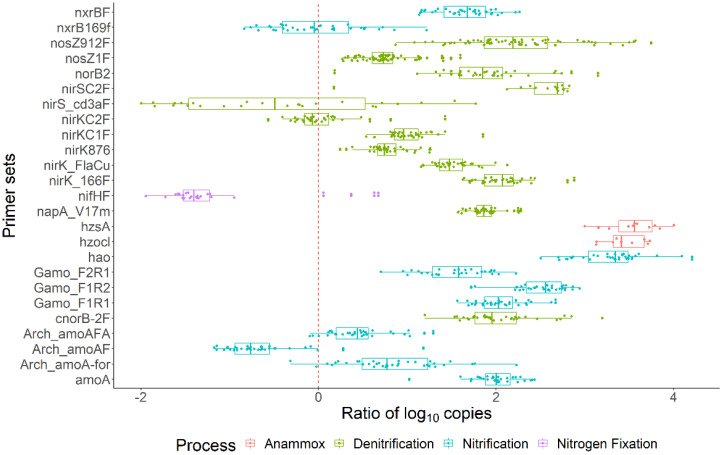
N cycle gene expression using ratios of log_10_ gene copies and transcript quantities. The processes include anammox, denitrification, nitrification, and nitrogen fixation.

To determine the prevalence of N-cycle genes, we asked what proportion of cells harbor specific genes, calculating the specific gene copy number per bacterial or archaeal 16SrRNA gene count. Using the 16SrRNA gene count as a proxy for cell number is clearly an oversimplification as the rRNA operon copy number varies between 1 and 15 across prokaryotes [52], so a 1.5 log_10_ range in accuracy must be assumed. Yet, the estimations based on this normalization indicate that the various nitrogen cycle genes occurred in 0.01 to 10% of bacteria (Figure 4). The data showed that most of the ammonia oxidation (*amoA*, *Gamo*) gene and transcript expression levels are similar across the bacterial community. In contrast, nitrogen fixation (*nifH*) and several denitrification-related genes (*nirS*, *nirK*, *nosZ*, etc.) have comparatively lower bacterial proportions. Intriguingly, N-cycle gene transcripts made up a smaller proportion of the overall transcriptional level, as measured by the 16SrRNA count. The DNA and RNA proportions shown in Figure 4 were derived using different denominators and cannot be directly compared, so we calculated the transcriptional activity of individual genes. In almost all cases, the mRNA levels were higher than the corresponding DNA copy numbers, indicating that these genes were being expressed strongly (Figure 5). Only *nxrB*, *nirS*, *nifH*, and *amoA* (archaeal) had a median ratio below zero, which signifies that they were poorly expressed. Collectively, these data show that most N-cycle genes were present in 0.1–1% of the prokaryotic community and that genes supporting nitrification, denitrification, and annamox were expressed strongly, while nitrogen fixation, as measured by *nifH,* was not. 

### 3.6. Bacterial Community and Functional Potential Were Unaffected by the Seasons

**Figure 6:**
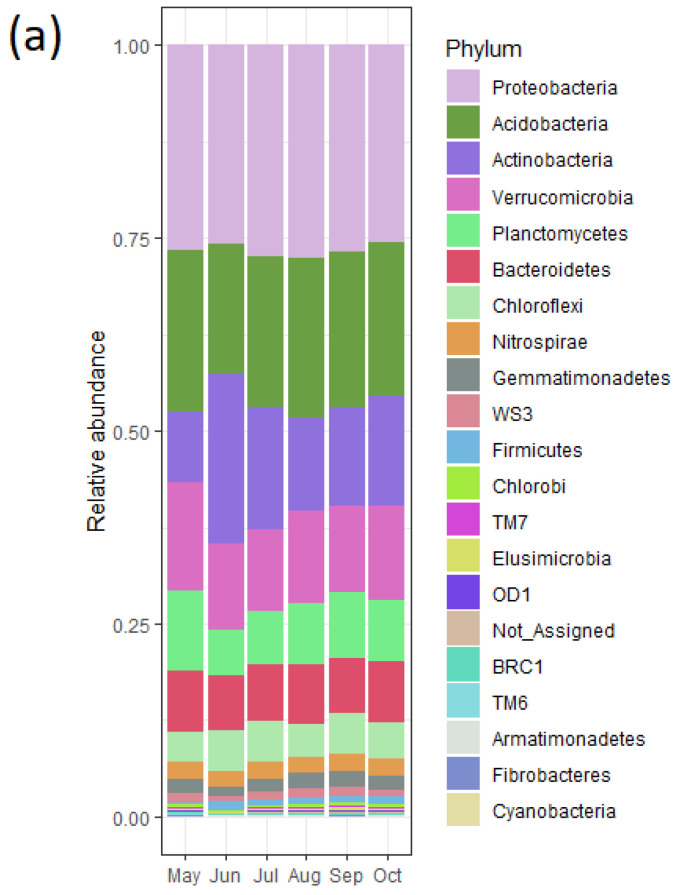

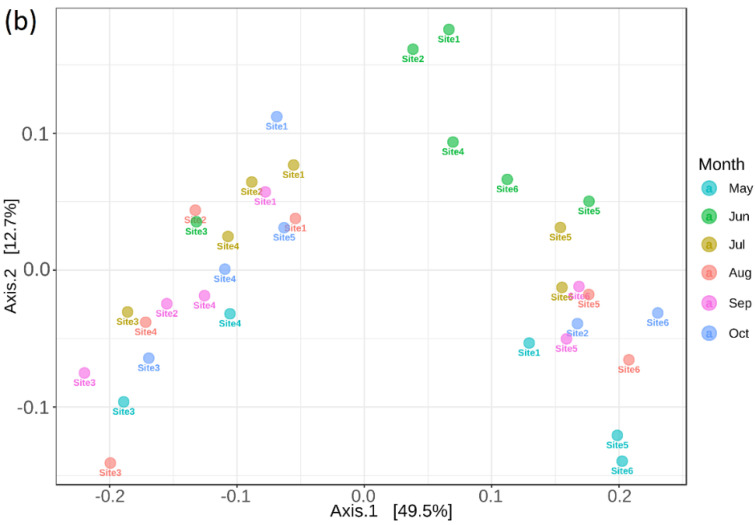
Bacterial community remains unchanged over seasons. (**a**) Stacked bar chart across the growing seasons; (**b**) PCoA showing beta-diversity using Bray–Curtis distance [PERMANOVA] F-value: 1.2403; R-squared: 0.17617; *p*-value < 0.217.

To explore the changes in the bacterial community structure across the seasons, we amplified the V_3_-V_4_ region of the bacterial 16S rRNA gene. Furthermore, functional predictions were extracted using PICRUSt2. The stacked bar-plot of the community structure across the sampling time points did not vary substantially (Figure 6a). In addition, alpha-diversity metrics—Shannon and Pielou evenness—show that the community indices were high and remained similar across all time points (Figure S5) (Kruskal–Wallis test). The beta-diversity analysis using Bray–Curtis dissimilarity analysis indicated that the bacterial diversity across the seasons was not unique but random ([PERMANOVA] F-value: 1.2403; R-squared: 0.17617; *p*-value < 0.217). The axes show that the sample points were not far apart in terms of dissimilarity (Figure 6b). The bacterial diversity may be somewhat unique by site as the data points for Site 3 clustered together. Furthermore, all data points for Sites 5 and 6 were close to each other. Upon deeper exploration of the bacterial taxonomy data, it was found that 157 taxa had a significantly different count (*p* < 0.05 by Kruska–Wallis test) for at least one time point (Figure S6). The relative abundance ranged between 0 to 6% and was distributed across 15 bacterial phyla.

**Figure 7:**
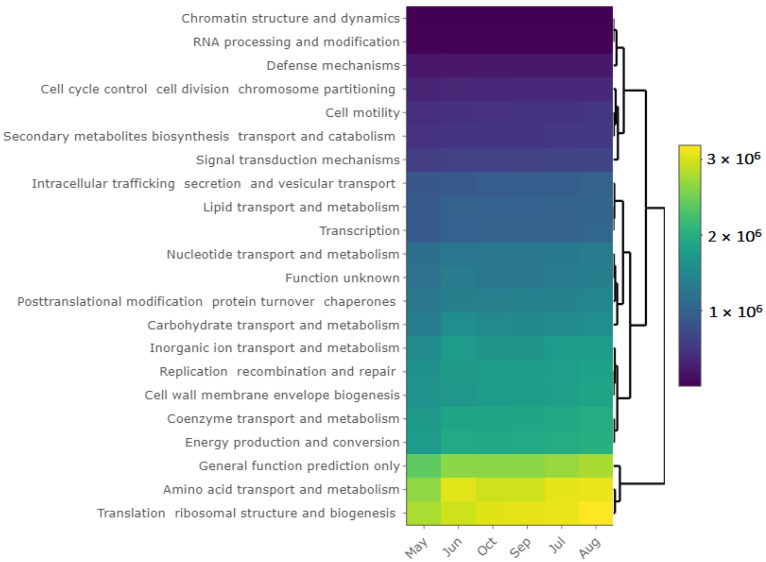
Heatmap showing Clusters of Orthologous Groups (COGs) of proteins derived using PICRUSt2 KEGG orthologs across sampling time points kept together based on *hclust* algorithm.

Several bacterial taxa 16S rRNA gene copies were high in the middle of the growing season (*p* < 0.05), for example, bacteria from the families *Sinobacteraceae*, *Syntrophobacteraceae*, *Pirellulaceae*, and a few uncharacterized (Class—S085), and the classes *Betaproteobacteria*, *Deltaproteobacteria*, and *Acidobacteria*. In contrast, copies of several bacterial taxa decreased in the middle of the growing season (*p* < 0.05), for example, the class *Acidimicrobia*, the families *Gaiellaceae*, *Chitinophagaceae*, and *Chthoniobacteraceae*, and the genus *Rhodoplanes*. Similar findings were obtained from the functional predictions. The most abundant groups of functions were related to amino acid transport and metabolism, general function prediction and translation, ribosomal structure, and biogenesis genes (Figure 7). In addition, low predicted counts were noted for secondary metabolites and motility-related genes. Despite some changes in the community composition, the bacterial diversity did not differ significantly across the growing seasons.

### 3.7. Bacterial Diversity, N Cycle Gene Expression, and Soil Chemistry

**Figure 8:**
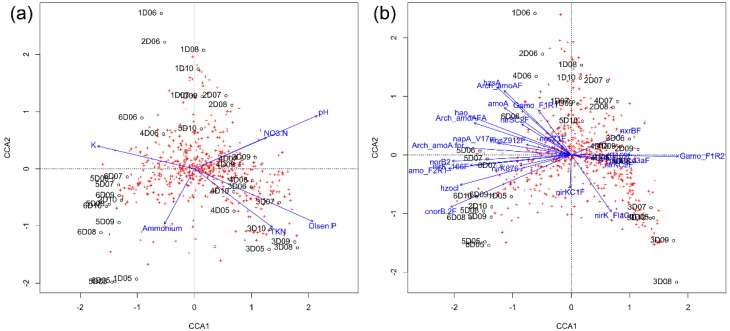
Bacterial beta–diversity and N cycle genes. (**a**) CCA showing relationship between OTU (red crosses) and soil chemical properties (blue lines). (**b**) CCA showing relationship between OTU (red crosses) and transcript copies of N cycle genes (blue lines).

We evaluated the bacterial diversity with the soil chemical properties and transcript quantities. All the chemical properties of the soil explored here contributed more or less to the slight variation seen in the bacterial community structure represented by the scatteredness of the OTUs (red pluses) and the data points (black dots) (Figure 8a and Supplementary Data S4). Soil pH and nitrate concentrations related more (Figure 2b, *p* < 0.05) with the variation in the months following July. Similarly, potassium was related to bacterial diversity in June and ammonium in May. A comparison of transcript quantities with the bacterial diversity shows that the gene transcripts related to ammonia oxidation, nitrite and nitrate reduction, and nitrous oxide reduction were largely responsible for the variation across some time points, especially for June (Figure 8b). Hence, the studied environmental factors contributed to the slight scatteredness seen in the bacterial taxa across the growing season.

## 4. Discussion

Late spring marks the onset of the growing season in upper mid-western prairies, with a significant increase in overall ecosystem activity, increasing the demand for available nitrogen in the soil. This study presents new insights into the seasonal dynamics of soil microbes in a natural grassland and points to an active microbial community involved in biochemical transformations required for N cycling. Furthermore, the study elucidates the soil bacterial community profiles and their predicted functional potential across the seasons. Using the NiCE chip, we found that N cycle gene presence and their respective transcript quantities did not vary significantly across seasons. Analysis of the 16S rRNA gene data and inferences from PICRUSt2 presented that neither bacterial communities nor their functional potential showed season-specific patterns. While the data refuted our working hypothesis that there would be seasonal differences in gene quantities and expression, it pointed to the consistent transformation of nitrogen intermediates and to stable bacterial communities in the prairie soil. Furthermore, the data suggested that the complexity of microbial N cycling mechanisms across growing seasons in the natural grassland system is high, as previously recorded in other grasslands [53].

### 4.1. N Cycle Genes and Transcript Quantities in a Heterogenous Grassland Are Stable

The bacterial and archaeal genes associated with N cycling did not show the anticipated seasonal changes. We expected that the activity and community composition of soil microbiota would be affected by root exudates [17] and would cause changes in gene copies and expression [10]. The grassland we studied was undulating with a consistent soil texture (Figure S1) and diverse plant composition (Figures S3 and S4). The clay-loam soil texture was predominant across sites, except Site 2, which is known to support high microbial biomass and abundance [54]. While some have reported changing N cycle genes in semi-arid grassland over time [55], our results indicate stable N cycling, contradicting our assumptions. Research in unfertilized, perennial grassland had similar results, with no changes over time [22]. There are some limitations of the NiCE chip employed here for the quantification of genes and transcripts. Some report the need for stringent criteria in the selection of primers, especially for nanoliter reaction volumes in the chip [56]. The efficiency of nucleic acid extraction from soil is not yet fully understood and could contribute to imprecise data. Yet, the microfluidic qPCR has been used to quantify multiple N cycle genes in wastewater [26] and soil [57] and can be adapted for the study of functional genes in different ecological settings.

The uniformity in the levels of bacterial and archaeal N-cycle gene and transcript presence across changing seasons in natural prairies suggests a buoyant but actively balanced system. Furthermore, the nitrogen cycle, when compared with bacterial transcript quantities, indicates a complete cycling, but with weak nitrogen fixation activity (Figure 2a). This suggests that nitrogen was not sufficiently replenished. Several reasons can be forwarded to elaborate this discrepancy. The primers for *nifH* are very specific and degenerate and that may have contributed to the inefficient amplification of the *nifH* cDNA copies. Earlier reports verify the difficulty in attaining complete coverage of the *nifH* gene population, with over 50 primer sets described in the literature [58]. There is simply no sufficiently conserved region in the prokaryotic *nifH* gene pool [59]. The degenerate PCR primers that must be used can cause template-specific bias and require challenging annealing temperatures [60]. Thus, our qPCR results may not be representative of the complete *nifH* gene pool in the prairie soil. Conversely, as N fixation is energy-intensive, and expression of *nifH* and other N fixation-related genes is strictly regulated at the transcription level [61], *nifH* transcript levels may have been as low as measured here. These results contradicted our assumptions, but the N cycle in this grassland may be unique and not broadly representative of grasslands overall. Yet, the question of how N is maintained in this ecosystem in the face of active N-efflux remains unanswered.

### 4.2. Nitrogen Cycle Genes and Soil Physio-Chemical Characteristics Are Strongly Correlated

Spearman correlation analysis showed that N cycle transcript quantities had strong correlations with each other and the soil chemical properties. It is known that microbial N cycling is affected by changes in N levels; for example, the addition of urea in soil caused increases in N cycling potential [62]. Here, we found that the Olsen phosphorus and pH are negatively correlated with processes related to the mobilization of N, either within the soil or removal through anammox, nitrate reduction, and denitrification (Figure 2b). Phosphorus content of soil had a similar correlation with *nifH* gene expression [63]. A negative correlation of ammonium concentration in soil with denitrification genes seems reasonable because a high expression of denitrifying genes would increase the removal of N from the soil. Moreover, the expression of genes towards anammox and denitrification were highly positively correlated with each other. Such correlations have been reported to enhance nitrogen removal in wastewater systems [64]. Thus, the chemical properties of soil were found to have a varied relationship with the N cycling genes [65,66]. Comparatively, soil chemical properties are correlated with the slight differences in the distribution of some bacterial taxa across the sampling time points (Figure 6b and Figure 8, Supplementary Data S3). Phosphorus [67] and pH [68] are known to be strongly correlated with bacterial diversity, and they can be used to estimate possible bacterial composition to some extent. Despite being responsible for variations in the bacterial community in certain soil types, soil chemical composition, except for soil nitrate and ammonium concentrations, did not change much with seasons in the natural grassland studied here (Figure S2). This suggests a community buffering system in soil that helps regulate the N cycle and the bacterial community overall, despite changes in nitrate and ammonium

### 4.3. Bacterial Communities and Functional Capabilities Are Synchronously Stable

Cool-season grasses dominate the prairie system (Figure S3), and they, along with forbs, are known to improve soil microbial biomass [69,70]. Prairie grass-root exudation undergoes seasonal shifts [13,14], suggesting bacterial community shifts. However, our soil microbial community shifted only slightly, and was not very responsive to the seasonal variation in root exudation or temporal shifts in soil chemistry (Figure 6a). A study using different bioenergy crops showed specificity in the soil N—transforming community due to changes in the type of bioenergy crops [71]. Furthermore, the spatial variation in our microbial communities was very weak. Similar results were found in a grassland system where plant diversity had a weak effect on microbial populations at small distances [72]. Soil prokaryotic microbiomes were stable across seasons in peatland soil [73] and in the sands of temperate and polar coastal regions [74]. The functional predictions made regarding the nitrogen cycle genes and overall bacterial community showed no variations across seasons, following May (Figure 1b and Figure 7). This is again in synchrony with the bacterial community composition, which was found to be associated with the soil properties and plant composition. Although some studies show that it underestimates the metabolic diversity [75], PICRUSt2 is an impressive tool to capture functional capability from 16S rRNA gene data [27], simplifying functional predictions in a time and cost effective way.

## 5. Conclusions

This study helps us understand the ecological role and dynamics of soil microbiota in a natural grassland across a complete growing season, i.e., May to October. Although the N cycle assays used here were pre-optimized for wastewater samples, 27 of 41 assays yielded reliable results. We found that the microbes actively partake in the N cycle but much less so for nitrogen fixation. This study successfully exploited the ease and efficiency of microfluidic qPCR for the rapid quantification of genes and transcripts. Furthermore, the 16S rRNA gene sequences revealed the presence of a highly diverse but stable microbial community unchanged across the seasons. Thus, we show that a natural grassland houses a consistent bacterial composition with stable N cycle genes and transcript quantities, previously assumed to be dynamic with environmental changes across the growing season.

## Data Availability

The raw data is available on NCBI’s Sequence Read Archive (SRA) database under BioProject: PRJNA803487.

## Supplementary Materials

The following supporting information can be downloaded at: https://www.mdpi.com/article/10.3390/microorganisms10050923/s1: Figure S1: Soil textures of the sampling sites; Figure S2: Distribution of plant groups across the six study sites; Figure S3: Plant species distribution across the study sites. The legume species are represented by asterisk (*) on the legend. The abbreviations represent: BRIN, *Bromus inermis*; POPR, *Poa pratensis*; PHAR, *Phalaris arundinacea*; CA, *Carex* sp.; NA, *Nassella* sp.; ANGE, *Andropogon gerardii*; SCSC, *Schizachyrium scoparium*; BOCU, *Bouteloua curtipendula*; DIOL, *Dichanthelium oligosanthes*; CIFL, *Cirsium flodmanii*; ASSP, *Asclepias* *speciose*; SOMI, *Solidago missouriensis*; HEMA, *Helianthus maximiliani*; ANCA, *Anemone canadensis*; SOCA, *Solidago canadensis*; GLLE, *Glycyrrhiza lepidota*; PH, *Physalis* sp.; AMCA*, *Amorpha canescens*; DAPU*, *Dalea purpurea*; PE*, *Pediomelum* sp.; RO, *Rosa* sp.; Figure S4: Soil chemical properties across sampling sites within the sampling time points with Kruskal–Wallis test results; Figure S5: Alpha-diversity of the bacterial communities across 6 months. (a) Shannon diversity and (b) Pielou Evenness.; Figure S6: Taxa differences across at least one time point across the seasons (*p* < 0.01). The names of the phyla shown here are based on the taxonomic profile downloaded from the Greengenes database, however, some of the phylum names have recently been changed [38]; Table S1. G-Block solutions; Table S2. Primers used for nitrogen cycle genes. Supplementary Data S3. R^2^ values of the qPCR assays. Supplementary Data S4. OTU table.
