# Supplementary material for: The Microbial Nitrogen Cycling, Bacterial Community Composition, and Functional Potential in a Natural Grassland Are Stable from Breaking Dormancy to Being Dormant Again
Source: Microorganisms. 2022 Apr 28;10(5):923. doi: 10.3390/microorganisms10050923 (PMC9148154; doi:10.3390/microorganisms10050923)
Supplement: Supplementary file 1 [file microorganisms-10-00923-s001.zip › Supplementary Figures and tables.pdf]

Supplemental

Supplemental figures

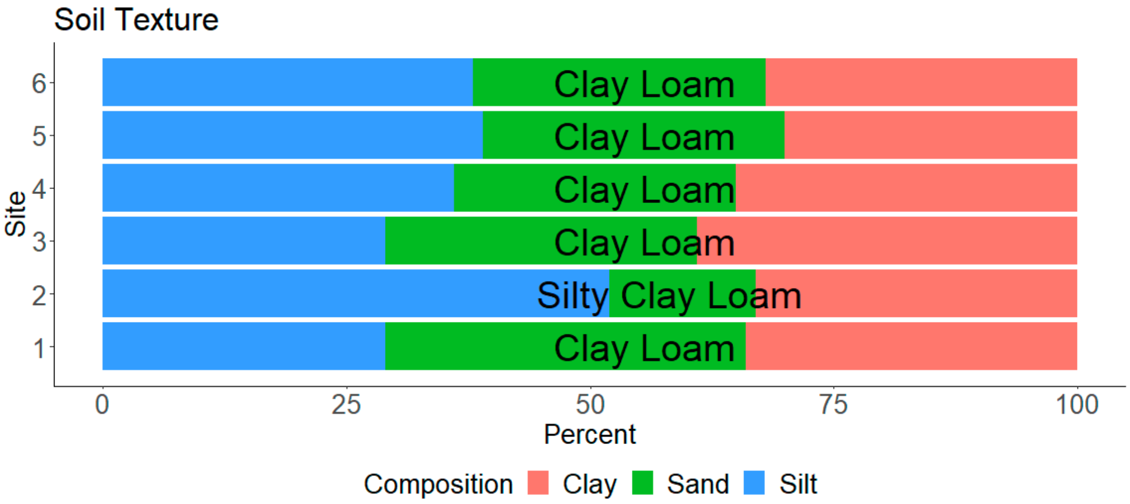

Figure S1: Soil textures of the sampling sites

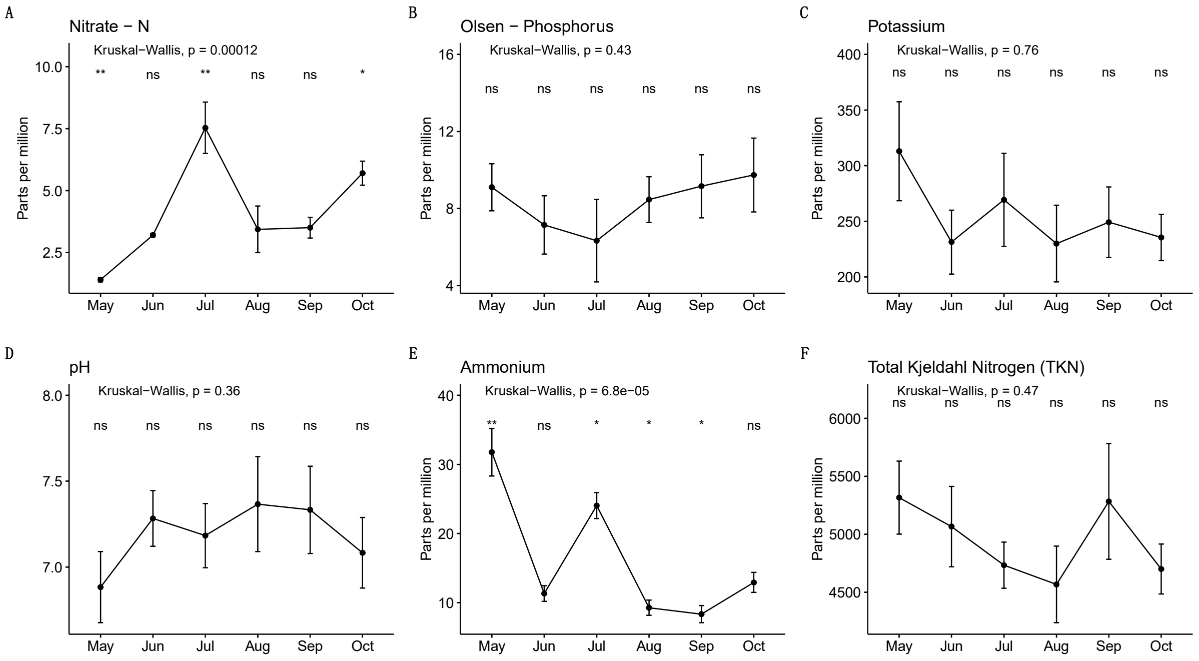

Figure S2: Soil chemical properties across sampling sites within the sampling time points with Kruskal-Wallis test results.

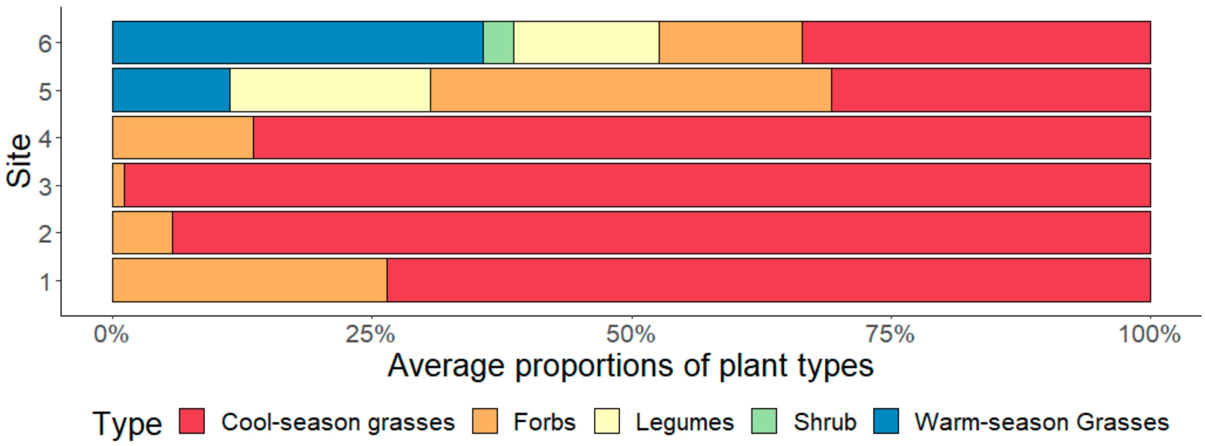

Figure S3. Distribution of plant groups across the six study sites.

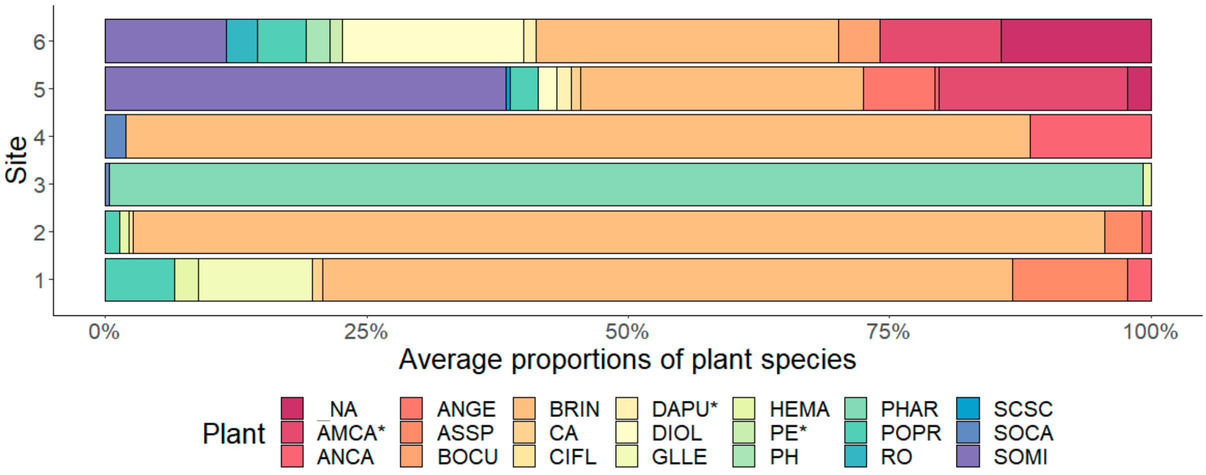

Figure S4. Plant species distribution across the study sites. The legume species are represented by asterisk (*) on the legend. The abbreviations represent: BRIN - *Bromus inermis*; POPR - *Poa pratensis*; PHAR - *Phalaris arundinacea*; CA – *Carex sp.*; NA – *Nassella sp.*; ANGE - *Andropogon gerardii*; SCSC - *Schizachyrium scoparium*; BOCU - *Bouteloua curtipendula*; DIOL - *Dichanthelium oligosanthes*; CIFL - *Cirsium flodmanii*; ASSP - *Asclepias speciose*; SOMI - *Solidago missouriensis*; HEMA - *Helianthus maximiliani*; ANCA - *Anemone canadensis*; SOCA - *Solidago canadensis*; GLLE - *Glycyrrhiza lepidota*; PH – *Physalis sp.*; AMCA* - *Amorpha canescens*; DAPU* - *Dalea purpurea*; PE* - *Pediomelum sp.*; RO – *Rosa sp.*

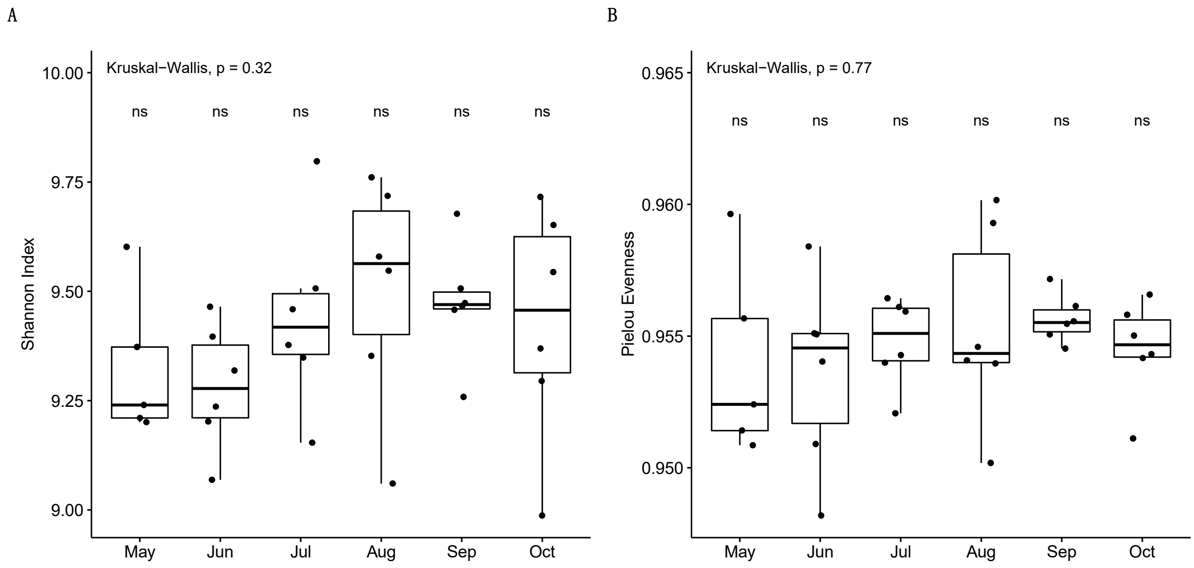

Figure S5. Alpha-diversity of the bacterial communities across 6 months (a) Shannon diversity, and (b) Pielou Evenness.

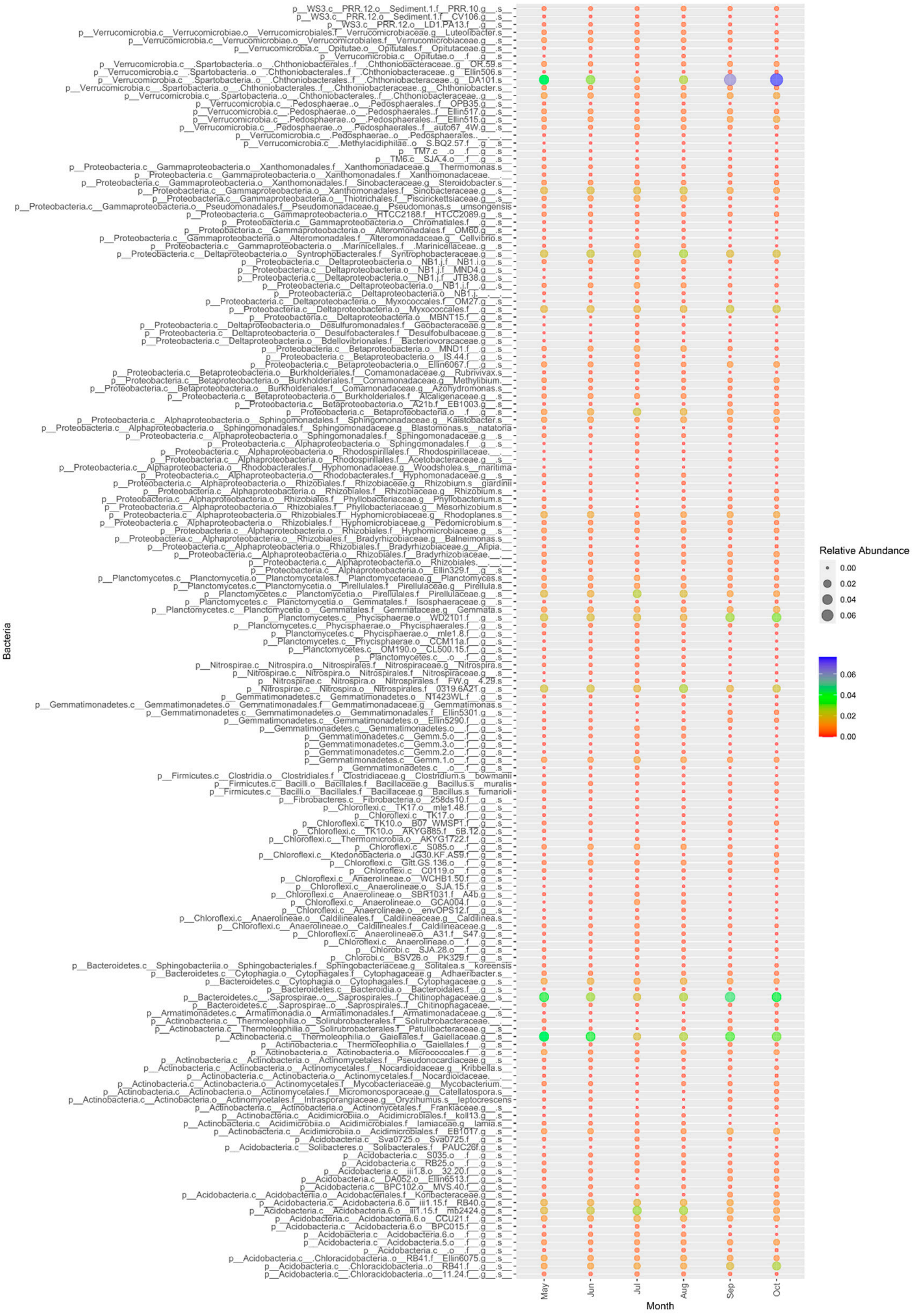

Figure S6: Taxa different across at least one time point across the seasons (p<0.01). The names of the phyla shown here are based on the taxonomic profile downloaded from the Greengenes database, however, some of the phylum names have recently been changed (Oren & Garrity, 2011).

Supplemental tables

Table S1. G-Block solutions

S.No.	Target	Type	S.No.	Target	Type
1	Bac_16S	gBlock	16	hzs	gBlock
2	Arc_16S	gBlock	17	hao	gBlock
3	nirK_1	gBlock	18	hzo	gBlock
4	nirK_2	gBlock	19	amoA_1	gBlock
5	nirS_1	gBlock	20	amoA_2	gBlock
6	norB_1	gBlock	21	amoA_3	gBlock
7	nosZ_1	gBlock	22	amoA_4	gBlock
8	nosZ_2	gBlock	23	amoA_5	gBlock
9	narG_2	gBlock	24	amoA_6	gBlock
10	norB_2	gBlock	25	nirK_3	gBlock
11	napA	gBlock	26	nirS_2	gBlock
12	nrfA	gBlock	27	nirS_3	gBlock
13	nifH	gBlock	28	narG_1	plasmid
14	nxrB_1	gBlock	29	nosZ_3	plasmid
15	nxrB_2	gBlock	30	comaA	gBlock

Table S2. Primers used for nitrogen cycle genes

Gene	Organism	Forward	Reverse	Assay No.	Assay ID
16S rRNA gene	Bacteria	515F	806R	1	1_16S
	Archaea	Archaea-F KO	Archaea-R KO	2	2_Arch_16S
*amo*A	γ-proteobacteria	Gamo172 F1	Gamo172 F1_R1	3	3_Gamo_F1R1
		Gamo172 F1	Gamo172 F1_R2	4	4_Gamo_F1R2
		Gamo172 F2	Gamo172 F2_R1	5	5_Gamo_F2R1
*amo*A	β-proteobacteria	amoA_F1	amoA_2R	6	6_amoA

Gene	Organism	Forward	Reverse	Assay No.	Assay ID
	Archaea	Arch-amoAF	Arch-amoAR	7	7_Arch_amoAF
		Arch-amoAFA	Arch-amoAR	8	8_Arch_amoAFA
		Arch-amoAFB	Arch-amoAR	9	9_Arch_amoAFB
		Arch-amoA-for	Arch-amoA-rev	10	10_Arch_amoA-for
hao/*hdh*	anammox bacteria	hzocl1F1	hzocl1R2	11	11_hzocl
	Proteobacterial AOB	haoF4	haoR2	12	12_hao
hzs	anammox bacteria	hzsA_1597F	hzsA1857R	13	13_hzsA
*nxr*B	Nitrobacter	NxrB 1F	NxrB 1R	14	14_nxrBF
	Nitrospira	nxrB169f	nxrB638r	15	15_nxrB169f
*nar*G	Bacteria	W9F	T38R	16	16_narG_W9F
		narG1960f	narG2650r	17	17_narG_1960f
*nrf*A	Bacteria	nrfAF2aw	nrfAR1	18	18_nrfA
*nap*A	Bacteria	V66	V67	19	19_napA_V66
		V17m	napA4r	20	20_napA_V17m
nirS	Bacteria	nirSCd3aF	nirSR3cd	21	21_nirS_cd3aF
		nirSC1F	nirSC1R	22	22_nirSC1F
		nirSC2F	nirSC2R	23	23_nirSC2F
		nirSC3F	nirSC3R	24	23_nirSC3F
*nir*K	Bacteria	FlaCu	R3Cu	25	25_nirK_FlaCu
		nirK876	nirK1040	26	26_nirK876
		nirKC1F	nirKC1R	27	27_nirKC1F
		nirKC2F	nirKC2R	28	28_nirKC2F
		nirKC4F	nirKC4R	29	29_nirKC4F
	AOB	nirK_166F	nirK_665R	30	30_nirK_166F
norB	denitrifier	norB2	norB6	31	31_norB2
		cnorB-2F	cnorB-6R	32	32_cnorB-2F
	Bacteria	qnorB2F	qnorB5R	33	33_qnorB2F-5R
		qnorB2F	qnorB7R	34	34_qnorB2F-7R
*nos*Z	denitrifier, clade I	nosZ1F	nosZ1R	35	35_nosZ1F
		nosZ-F-1181	nosZ-R-1880	36	36_nosZ-F-1181
	denitrifier, clade II	nosZ-II-F	nosZ-II-R	37	37_nosZ-II-F
		NosZ912F	NosZ1853R	38	38_nosZ912F
nifH	Bacteria	nifHF	nifHR	39	39_nifHF
		IGK3	DVV_correct	40	40_nifH_IGK3
comaA	comammox	comaA-244F	comaA-659R	41	41_comaA-244F
